# Simultaneous bilateral testicular metastases from renal clear cell carcinoma: A case report and review of the literature

**DOI:** 10.3892/ol.2014.1830

**Published:** 2014-01-27

**Authors:** SHINGO MORIYAMA, HIDEKI TAKESHITA, AKIKO ADACHI, YOSHIAKI ARAI, SAORI HIGUCHI, TAKUO TOKAIRIN, KOJI CHIBA, KOJI NAKAGAWA, AKIRA NORO

**Affiliations:** 1 Department of Urology, Saitama Red Cross Hospital, Saitama, Saitama 338-8553, Japan; 2 Department of Pathology, Saitama Red Cross Hospital, Saitama, Saitama 338-8553, Japan; 3 Department of Urology, Nishi-Ohmiya Hospital, Saitama City, Saitama 330-0856, Japan; 4 Department of Surgery, Saitama Red Cross Hospital, Saitama City, Saitama 338-8553, Japan

**Keywords:** renal cell carcinoma, metastasis, testicular neoplasm

## Abstract

Metastasis from renal cell carcinoma (RCC) to the testis is rare. This case report presented an extremely rare case of simultaneous bilateral testicular metastases from RCC in a 65-year-old man who had experienced indolent scrotal enlargement over a period of several months. Scrotal ultrasonography showed 4.0- and 2.0-cm-sized masses in the left and right testes, respectively. Contrast-enhanced computed tomography identified multiple tumors in the kidneys, the pancreas and the left adrenal gland. Left orchiectomy and pathological examination were performed and indicated testicular metastasis from clear cell RCC. The patient underwent complete surgical resection of all residual lesions. Postoperative follow-up examination without adjuvant therapy identified no recurrence over 11 months. This study also reviewed existing literature and determined that retrograde venous spread from the primary kidney tumor to the testis may be an important pathway for testicular metastasis from RCC. In conclusion, RCC can result in testicular metastases not only unilaterally, but also bilaterally, as was observed in the present case.

## Introduction

Secondary neoplasms of the testis are rare, with an incidence of 0.9% in all testicular tumors according to a previous German survey (1). The most common location of the primary neoplasm is the prostate, followed by the gastrointestinal tract, lungs and kidneys (2–4). Renal cell carcinoma (RCC) commonly results in metastases to various organs. Although RCC metastasis is frequently observed in the lungs, lymph nodes, bones, liver and the brain, it is rarely identified in the testes (5,6). Several cases of unilateral testicular metastasis from RCC have been reported (1,7). Dieckmann *et al* (1) reported 13 cases of unilateral testicular metastases with detailed clinical information. They speculated that testicular metastases have left lateral dominance by analysing unilateral cases. However, simultaneous bilateral testicular metastases have not yet been determined. This report presented an extremely rare case of pathologically proven simultaneous bilateral testicular metastases from RCC. In addition, this study reviewed the previously reported cases of testicular metastases from RCC.

## Case report

**Figure 1:**
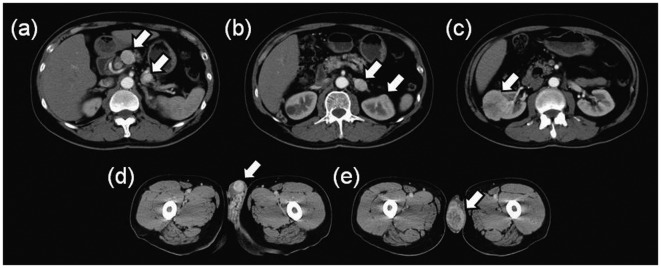
Abdominal computed tomography showing multiple tumors in various organs (arrows), including the (a) pancreas, (b) left adrenal gland and left kidney, (c) right kidney, (d) right testis and (e) left testis.

A 65-year-old man was referred to the Saitama Red Cross Hospital (Saitama, Japan) with a complaint of indolent left scrotal enlargement over several months. Physical examination revealed a stony, hard, hen’s egg-sized mass in the left scrotum. A normal-sized testis with a small nodule was observed in the contralateral scrotum. Superficial lymph nodes were not palpable. Serum levels of α-fetoprotein, β-human chorionic gonadotropin and soluble interleukin-2 receptor were all within normal limits. Scrotal ultrasonography revealed a 4.0×3.3-cm mass in the left testis and a 2.0×1.7-cm mass in the right testis. Contrast-enhanced computed tomography of the abdomen identified multiple tumors in the two kidneys, the pancreas and the left adrenal gland, in addition to the testes (Fig. 1). Imaging studies did not show metastasis in other regions, such as the bone, lungs and brain.

**Figure 2:**
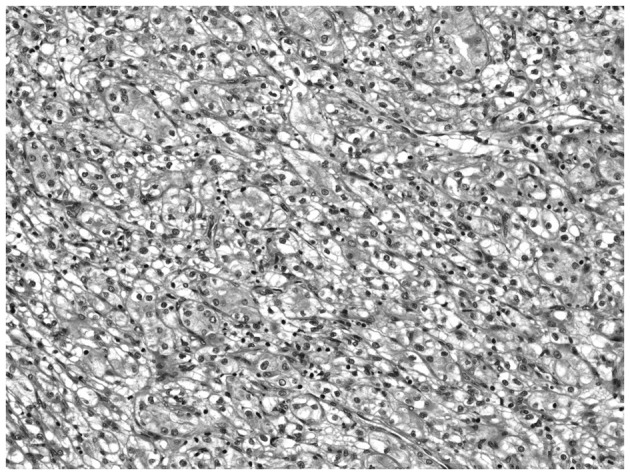
Microscopic section of the left testicular tumor shows a typical pattern of clear cell renal carcinoma. All the disseminated lesions, which were surgically resected, showed the same findings (hematoxylin and eosin, magnification, ×200).

We performed left orchiectomy for pathological diagnosis. The resected testis contained a yellowish-white tumor with clear margins accompanied by parenchymal hemorrhage. Pathological examination revealed that the tumor cells had small, slightly oval nuclei with optically clear cytoplasm and were arranged in nests separated by a rich network of sinusoidal vascular channels (Fig. 2). These results were compatible with a diagnosis of metastasis from clear cell RCC. The patient was diagnosed with right RCC that was metastasizing to the contralateral kidney and adrenal gland, the pancreas and the testes (staging, cT1bN0M1).

All disseminated tumors were surgically resectable and the patient’s general condition was good. Therefore, the patient underwent partial pancreatectomy, left adrenalectomy and left partial nephrectomy, followed by right radical nephrectomy and right partial orchiectomy. Complete surgical resection was achieved. The pathological findings of the resected tumors were compatible with metastases from the right RCC (clear cell carcinoma, Grade II, pT1b). Postoperative follow-up examination without adjuvant therapy showed no recurrence for 11 months. The patient provided written informed consent.

## Discussion

Secondary neoplasms of the testis are rare with a reported incidence of testicular metastasis of 0.02% (8) and 0.06% (2)at autopsy and testicular metastasis accounted for 0.9% of all types of testicular tumors (1). Table I summarized 30 cases of testicular metastasis from RCC. Bilateral testicular metastasis from RCC has not been previously reported. However, several cases of bilateral testicular metastases from prostate cancer (9) and colorectal cancer (10) have been determined.

Although RCC commonly results in metastases to various organs, it rarely spreads to the testes. The testes are regarded as a ‘tumor sanctuary’, as it has been hypothesized that tumor cells are not able to grow easily in that environment. The relatively low temperature of the scrotum could provide unacceptable conditions for the establishment of metastatic tumor cells (5). Additionally, the presence of the blood-testis barrier formed by Sertoli cells, which physiologically aims to protect spermatozoa, may also play an indirect role in the prevention of testicular metastasis (6).

This study also searched previous medical literature using the Medline/PubMed databases and identified 30 reported cases of unilateral testicular metastasis from RCC, excluding autopsy cases (Table I). Of the 30 cases, 15 were of left testicular metastases (50%) and 15 were of right testicular metastases (50%). Although the left side is thought to be involved more often than the right side (1,11), we did not observe any particular laterality of testicular metastasis. The association between primary kidney tumors and the testis totaled 18 ipsilateral metastases and nine contralateral metastases. Due to the tendency of metastasis from the kidney to the testis on the same side, there may be important spreading routes between the kidney and the testis. One of main routes could be a retrograde venous spread via the spermatic vein (1,2,8). In the present case, the primary kidney and the larger testicular metastasis had the same laterality; therefore, superiority of ipsilateral metastasis was suggested.

In conclusion, to the best of our knowledge, this study was the first to present an extremely rare case of simultaneous bilateral testicular metastases from RCC. Following a review of the current literature, ipsilateral testicular metastasis from RCC is more frequent and, thus, retrograde venous spread via the spermatic vein may be one of the main pathways of testicular metastasis from RCC. As demonstrated in this case, RCC can result in testicular metastasis, not only unilaterally but also bilaterally.

## Figures and Tables

**Table I:** Testicular metastasis from renal cell carcinoma: review of the literature.

				Laterality			
					
Case no.	Author	Year	Age, years	Testis	Kidney	Association between the testis and the kidney	Solitary or multiple metastases
1	Bandler and Roen	1946	47	R	R	Ipsilateral	Solitary
2	Tuchschmid	1965	58	L	L	Ipsilateral	Solitary
3	Hanash *et al*	1969	70	R	R	Ipsilateral	NA
4	Talerman and Kniestedt	1974	68	L	L	Ipsilateral	Solitary
5	Nataf *et al*	1975	64	L	R	Contralateral	Solitary
6	Nataf *et al*	1975	55	R	L	Contralateral	Multiple
7	DeBre *et al*	1980	63	L	R	Contralateral	Solitary
8	Post and Kassis	1980	64	L	L	Ipsilateral	Solitary
9	Minervini *et al*	1984	56	L	L	Ipsilateral	Solitary
10	Yano *et al*	1985	62	L	L	Ipsilateral	Multiple
11	Ishizuka *et al*	1986	71	L	L	Ipsilateral	Multiple
12	De Riese *et al*	1986	60	L	L	Ipsilateral	Multiple
13	Dieckmann *et al*	1988	73	L	L	Ipsilateral	Multiple
14	Indudhara *et al*	1990	67	L	L	Ipsilateral	Solitary
15	Daniels *et al*	1991	87	R	L	Contralateral	Solitary
16	Ribalta *et al*	1993	62	R	R	Ipsilateral	Multiple
17	Blasco *et al*	1994	72	L	L	Ipsilateral	Solitary
18	Lauro *et al*	1998	56	R	L	Contralateral	Solitary
19	Steiner *et al*	1999	66	L	R	Contralateral	Solitary
20	Nabi *et al*	2001	60	R	L	Contralateral	Solitary
21	Datta *et al*	2001	81	R	NA	NA	Multiple
22	Datta *et al*	2001	67	L	R	Contralateral	Solitary
23	Datta *et al*	2001	85	R	R	Ipsilateral	Solitary
24	Datta *et al*	2001	53	R	NA	NA	Multiple
25	Marquez *et al*	2001	65	R	R	Ipsilateral	Solitary
26	Nemoto *et al*	2007	56	R	NA	NA	Multiple
27	Camerini *et al*	2007	46	R	R	Ipsilateral	Multiple
28	Llarena *et al*	2008	57	R	R	Ipsilateral	Multiple
29	Schmorl *et al*	2008	66	R	R	Ipsilateral	Multiple
30	Hai-yang *et al*	2010	70	L	R	Contralateral	Multiple
31	Present case	2013	65	Bilateral	R	Bilateral	Multiple

R, right; L, left; NA, not available.

## References

[1] Dieckmann KP, Düe W, Loy V (1988). Intrascrotal metastasis of renal cell carcinoma. Case reports and review of the literature. Eur Urol.

[2] Pienkos EJ, Jablokow VR (1972). Secondary testicular tumors. Cancer.

[3] Dutt N, Bates AW, Baithun SI (2000). Secondary neoplasms of the male genital tract with different patterns of involvement in adults and children. Histopathology.

[4] Haupt HM, Mann RB, Trump DL, Abeloff MD (1984). Metastatic carcinoma involving the testis. Clinical and pathologic distinction from primary testicular neoplasms. Cancer.

[5] Blefari F, Risi O, Pino P (1992). Secondary tumors of testis: two rare cases and review of the literature. Urol Int.

[6] Camerini A, Tartarelli G, Martini L, Donati S, Puccinelli P, Amoroso D (2007). Ipsilateral right testicular metastasis from renal cell carcinoma in a responder patient to interleukine-2 treatment. Int J Urol.

[7] Datta MW, Ulbright TM, Young RH (2001). Renal cell carcinoma metastatic to the testis and its adnexa: a report of five cases including three that accounted for the initial clinical presentation. Int J Surg Pathol.

[8] Hanash KA, Carney JA, Kelalis PP (1969). Metastatic tumors to testicles: routes of metastasis. J Urol.

[9] Giannakopoulos X, Bai M, Grammeniatis E, Stefanou D, Agnanti N (1994). Bilateral testicular metastasis of an adenocarcinoma of the prostate. Ann Urol.

[10] Hatoum HA, Abi Saad GS, Otrock ZK, Barada KA, Shamseddine AI (2011). Metastasis of colorectal carcinoma to the testes: clinical presentation and possible pathways. Int J Clin Oncol.

[11] Nabi G, Gania MA, Sharma MC (2001). Solitary delayed contralateral testicular metastasis from renal cell carcinoma. Indian J Pathol Microbiol.

